# Cross-reactive carbohydrate determinant interference in cellulose-based IgE allergy tests utilizing recombinant allergen components

**DOI:** 10.1371/journal.pone.0231344

**Published:** 2020-04-23

**Authors:** Edsel Sinson, Camille Ocampo, Cindy Liao, Steven Nguyen, Lauren Dinh, Kelline Rodems, Eric Whitters, Robert G. Hamilton

**Affiliations:** 1 HYCOR Biomedical LLC, Garden Grove, California, United States of America; 2 Department of Medicine, Johns Hopkins University School of Medicine, Baltimore, Maryland, United States of America; Harvard Medical School, UNITED STATES

## Abstract

**Background:**

Cross-reactive carbohydrate determinant (CCD) structures found in plant and insect glycoproteins are commonly recognized by IgE antibodies as epitopes that can lead to extensive cross-reactivity and obscure in vitro diagnostic (IVD) serology results. With the introduction of component resolved diagnosis (CRD), recombinant non-glycosylated components have been utilized to mitigate the risk of CCD-specific IgE (sIgE) detection. However, a recent study has shown that CCD-sIgE may bind directly to the cellulose solid phase matrix used in certain *in vitro* diagnostic assays, eliminating the advantage of CRD over traditional extract-based testing. The aim of this study is to further investigate the prevalence of CCD-sIgE interference on a commonly-used *in vitro* sIgE automated platform which employs a cellulose-based matrix to immobilize CCD-free recombinant components.

**Methods:**

Sera from patients sensitized to peanut, silver birch, and/or timothy grass were analyzed for CCD-sIgE reactivity on ImmunoCAP/Phadia and NOVEOS autoanalyzers against the MUXF3 carbohydrate component. Positive CCD-sIgE sera were further analyzed against non-glycosylated recombinant components bound to the ImmunoCAP solid phase in the absence and presence of a soluble CCD inhibitor. For comparison, sera were then analyzed on NOVEOS, a non-cellulose based automated sIgE assay.

**Results:**

Sera from 35% of the sensitized population tested in this study were positive (≥0.35 kU/L) for CCD-sIgE. Of those positives, 17% resulted in CCD-sIgE-positive (false positive) results on ImmunoCAP using non-glycosylated allergosorbents that were negative on NOVEOS. Sera producing false-positive results on ImmunoCAP had varying levels of CCD-sIgE from 0.67 kU/L to 36.52 kU/L. The incidence of CCD interference was predominantly delimited to low-positive IgE results (0.35 kU_A_/L– 3.00 kU_A_/L).

**Conclusion:**

Falsely elevated diagnostic allergen-sIgE results can commonly occur due to the presence of CCD-sIgE using assays that employ a carbohydrate matrix-based allergosorbent. Even the use of non-glycosylated recombinant allergenic components coupled to cellulose matrices do not reduce their risk of detection. The risk of CCD interference that compromises quantitative IgE results can be mitigated by the addition of a soluble CCD inhibitor to positive CCD-sIgE containing sera or by alternatively using a non-cellulose based sIgE assay, such as the NOVEOS assay.

## Introduction

Glycoproteins found in plants and insects display structural homology across taxonomically diverse allergenic sources due to the presence of complex asparagine-linked oligosaccharides known as N-glycans.[1–3] More specifically, it is the presence of a core α1,3-linked fucose or a β1,2-linked xylose that represent common post-translational modifications of glycoproteins in these species and are the key elements of the widespread carbohydrate pan-epitope structure.[1,4–7] N-glycans with this epitope are known as cross-reactive carbohydrate determinants (CCDs) which contain core modifications that differ from those found in human glycoproteins. Thus, these can be viewed by the human immune system as foreign and, in some individuals, may elicit the production of IgE antibodies.[1]

IgE antibodies reactive with CCD epitopes are believed to have limited or no clinical significance partly due to their low avidity and marginal biological activity.[8,9] When detected by *in vitro* diagnostic (IVD) assays, CCD reactive-IgE antibodies neither predicts the development of clinical symptoms upon allergen exposure nor does it associate with disease severity.[10–12] CCD reactivity, however, can impact the diagnostic accuracy of the quantitative measurement of IgE antibodies in a patient’s serum analysis.

Approximately 30% of the allergic population sera contain CCD-sIgE.[13,14] Component resolved diagnosis (CRD), using recombinant allergens with no apparent glycosylation, has therefore been recommended to reduce the risk of obtaining inaccurate results.[15,16] CRD’s ability to discriminate between various aspects of clinical disease results in an improved diagnostic specificity and sensitivity. This leads to more effective therapeutic strategies and accurate predictions of allergic disease severity.[1,17–19]

Currently, the most widely used single complexity allergen-specific IgE assay utilizing CRD is the ImmunoCAP (Thermo Scientific, ImmunoDiagnostics, Uppsala, Sweden) with over 100 components available for testing. However, a recent study has shown that the ImmunoCAP polymerized cellulose matrix used to bind allergenic proteins contains CCD epitopes that are recognizable by IgE antibodies.[20] This means that the CCD-sIgE of a patient tested against an advertised “CCD-free recombinant protein,” such as rAra h 8, may recognize N-glycans present on the cellulose matrix; which would result in an increased rate of false-positive results, and at a minimum, reduce confidence in the accuracy of results generated on these cellulose-based assays. With many clinicians unaware of this issue, the interpretation of recombinant CRD results may lead to an incorrect diagnosis if the patient sera contains levels of CCD-sIgE.

The aim of this study was to further investigate the prevalence of CCD interference on the ImmunoCAP allergen-specific IgE assay when utilizing CCD-free recombinant proteins and to offer suitable alternatives for specific IgE testing when the need to mitigate the detection of CCD-sIgE is required.

## Materials and methods

### Study population

In total, 204 serum samples were selected for this study based on a positive (≥ 0.35 kU_A_/L) sIgE result to peanut, silver birch, and/or timothy grass when tested on ImmunoCAP. Of these samples, 124 were monosensitized to only one allergen while the remaining 80 sera were multi sensitized to more than one allergen. This study used leftover specimens, that is, remnants of specimens collected for routine clinical care or analysis in the United States that would have been discarded. The specimens were received as deidentified samples i.e., the identity of the subject is not known to and may not readily be ascertained by the investigator or any other individuals associated with the investigation, including the sponsor, with no additional information of clinical reactivity to the tested allergens available.

### CCD-specific IgE testing

In order to conserve patient sera volume for the additional testing required for positive CCD-sIgE samples, screening of all 204 samples was performed on the NOVEOS specific IgE assay (HYCOR Biomedical, California, United States) against the MUXF3 carbohydrate component (allergen code O214). The NOVEOS Immunoanalyzer was the preferred method for CCD-sIgE testing given the 4 μL sample volume requirement for each sIgE determination compared to ImmunoCAP sIgE per test 40 μL requirement. A small subset of patients (n = 32) with adequate volume was re-tested with the ImmunoCAP CCD test (allergen code O214) as a reference method as presented in supplemental Fig 1.

**Fig 1:**
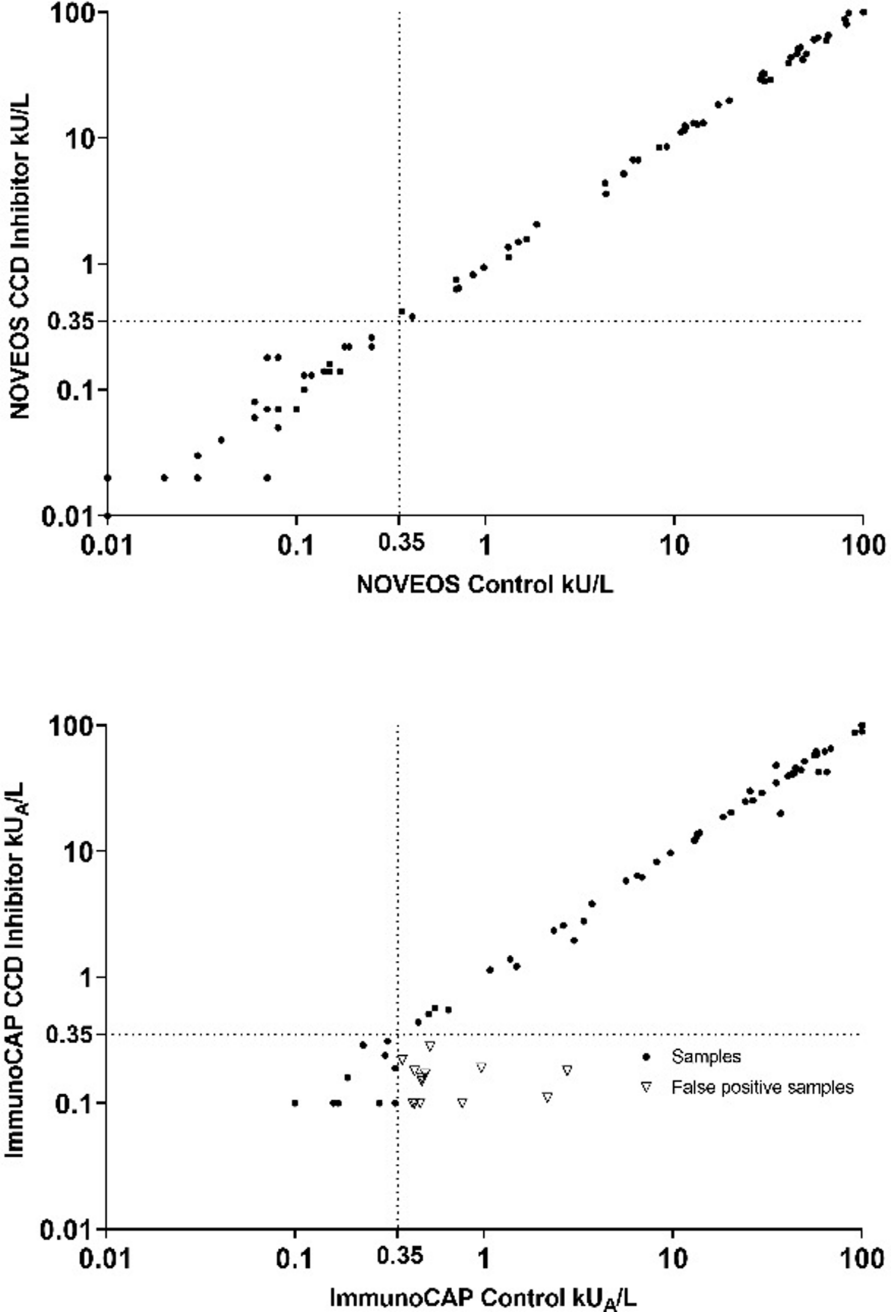
Correlation of patient specific IgE results with and without CCD inhibitor on NOVEOS(a) and ImmunoCAP(b) when tested against CCD-free recombinant components.

### Specific IgE recombinant component testing

Sera from patients that are positive for CCD-sIgE with known sensitivity (≥ 0.35 kU_A_/L) to peanut were tested against rAra h 8 (PR-10). Those with known sensitivity to silver birch were tested against rBet v 1 (PR-10), and sera exhibiting a known sensitivity to timothy grass were analyzed against rPhl p 1 (b-Expansin) with and without CCD inhibitor on NOVOES and ImmunoCAP. These recombinant allergenic molecules were produced with an absence of glycosylation and produced in an E. coli strain carrying a cloned cDNA encoding the allergen component of interest.

Testing with and without competitive CCD inhibition was performed within the same run and day on ImmunoCAP in order to measure the rate of false-positives due to CCD-sIgE binding to the cellulose solid phase. Since recombinant non-glycosylated components were the sole allergens used in this study, a reduction in the concentration of specific IgE with inhibitor compared to the control (absence of inhibitor) indicates the presence of CCD-sIgE binding to the cellulose matrix of the ImmunoCAP assay.

Specific IgE testing was also performed on NOVEOS to demonstrate the performance of patients with and without CCD inhibitor on an immunoanalyzer where the sIgE assay does not use N-glycans or cellulose on its solid phase. NOVEOS utilizes paramagnetic microparticle beads as the solid phase to which the allergenic proteins are bound. As of this publication date, NOVOES components rAra h 8, rBet v 1, and rPhl p 1 are CE marked and currently unavailable in the U.S.

### Inhibition of CCD-sIgE

Soluble CCD competitive inhibition was performed on all sera that had a positive CCD-sIgE result (≥ 0.35 kU/L) with the NOVEOS assay. A commercially available semi-synthetic inhibitor (www.proglycan.com, Vienna, Austria) consisting of purified MUXF3 glycopeptides obtained from bromelain and coupled to human serum albumin was used to block IgE antibodies against CCDs. The inhibitor, with a starting concentration of 1 mg/mL, was added to each serum to obtain a final concentration of 20 μg/mL; e.g., 4 μL of CCD inhibitor was added to 200 μL of serum. The inhibitor-serum mix was then let to incubate for a minimum of one hour at room temperature.

## Results

### Prevalence of CCD-sIgE in patients sensitized to timothy grass, silver birch, and peanut

The present study analyzed 204 patients; approximately 35% (n = 72) were positive for CCD-sIgE when tested on NOVEOS (Table 1). The highest rate of positivity were patients sensitized only to peanut (46%), followed by patients sensitized to more than one allergen (44%). Lower rates were observed with patients sensitized to only silver birch (19%) and timothy grass (17%). The average CCD-sIgE concentration for these patients was 4.46 kU/L (95% CI: 2.25 kU/L– 6.67 kU/L, min: 0.35 kU/L, max: 69.70 kU/L). Due to insufficient serum, 8 of the 72 positive-CCD patients were not included in additional specific IgE testing.

**Table 1:** Quantitative levels of CCD-sIgE in a sensitized population to peanut, timothy grass, and silver birch.

	CCD IgE Positive		
*Group*	n =	%	Average CCD IgE Concentration kU/L (95% CI)
Total (n = 204)	72	35.3%	4.46 (2.25–6.67)
*Group by allergen*			
Peanut (n = 52)	24	46.2%	2.79 (1.46–4.11)
Silver Birch (n = 42)	8	19.0%	9.76 (2.97–16.55)
Timothy Grass (n = 30)	5	16.7%	6.6 (2.96–10.24)
Multi sensitized (n = 80)	35	43.8%	4.09 (2.21–5.97)

### False-positives due to CCD-sIgE interference

Samples with known reactivity to a given extract from the CCD-positive population were tested with recombinant components (presumably N-linked glycan free) with and without CCD inhibitor (Fig 1). As some patients were tested with two or more components due to their multi sensitization, the 64 positive CCD-sIgE patients resulted in a total of 92 recombinant sIgE test results on NOVEOS and ImmunoCAP. When performed on ImmunoCAP, 15.2% (14/92) of all tests resulted in a false-positive result. The rate of false-positive results (e.g. ≥0.35 kU_A_/L) by component analysis was 19.0% (8/42) for rAra h 8, 12.9% (4/31) for rBet v 1, and 10.5% (2/19) for rPhl p 1. Quantitatively, the specific IgE results from these false-positives ranged from 0.37 kU_A_/L to 2.76 kU_A_/L without a CCD inhibitor but converted to negative (<0.35 kU_A_/L) when the CCD inhibitor was pre-incubated with the same serum.

**Fig 2:**
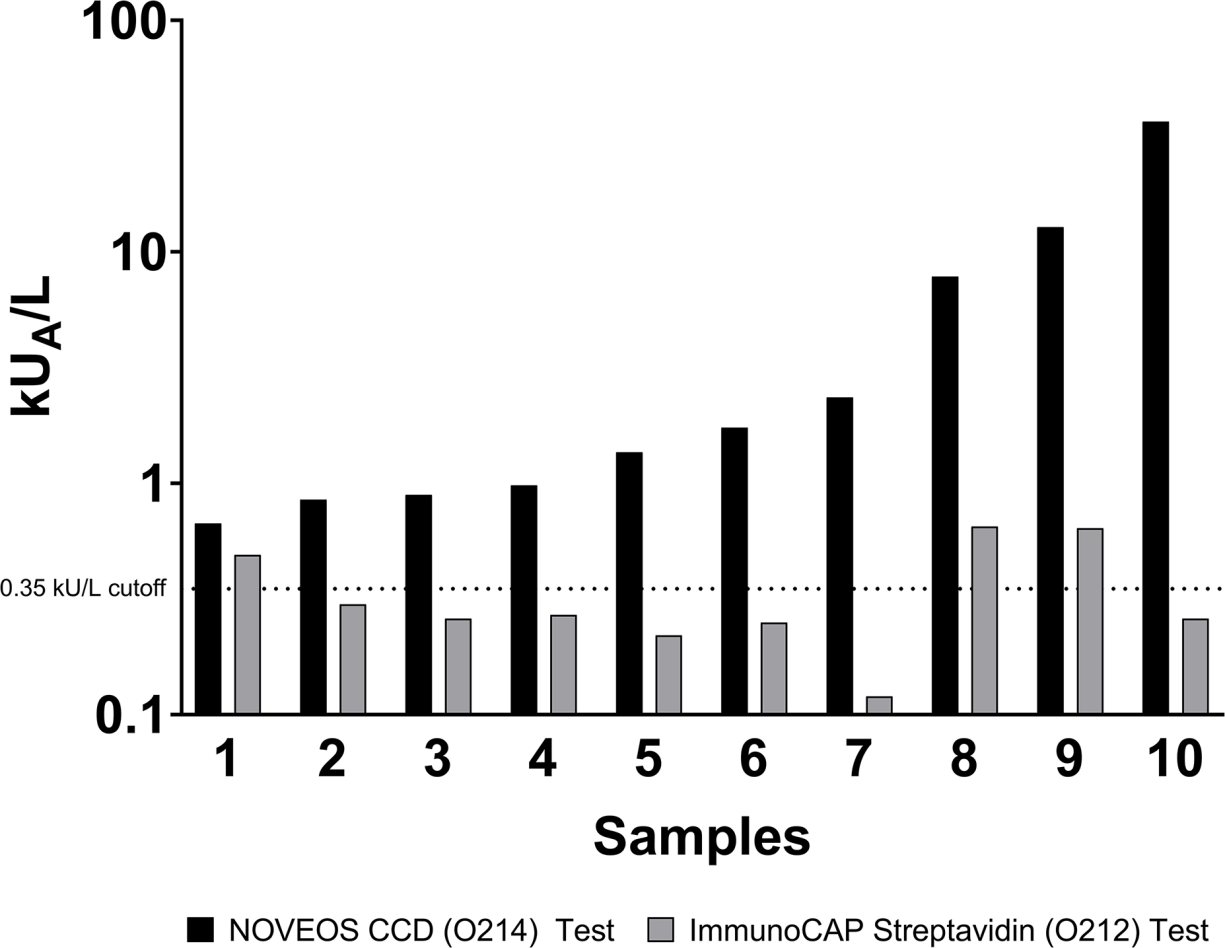
Positive CCD-sIgE patients resulting in false-positive results were tested against ImmunoCAP streptavidin test (O212) with no allergenic proteins present. A single patient was not included due to insufficient volume.

When analyzed by sample population, 17.2% (11/64) of CCD-positive patients accounted for all of the false-positive results observed on ImmunoCAP and displayed an average CCD-sIgE reactivity of 6.99 kU/L (95% CI: 0.64 kU/L– 13.3 kU/L, min: 0.67 kU/L, max: 36.52 kU/L). Even when tested against the ImmunoCAP streptavidin test (O212) which is devoid of any allergens, sIgE levels between 0.12 kU_A_/L to 0.65kU_A_/L were still observed across the false-positive samples. This means each sample’s CCD-sIgE can bind directly to the cellulose matrix of the allergosorbent contributing to inaccurate elevated recombinant component results (Fig 2).

**Fig 3:**
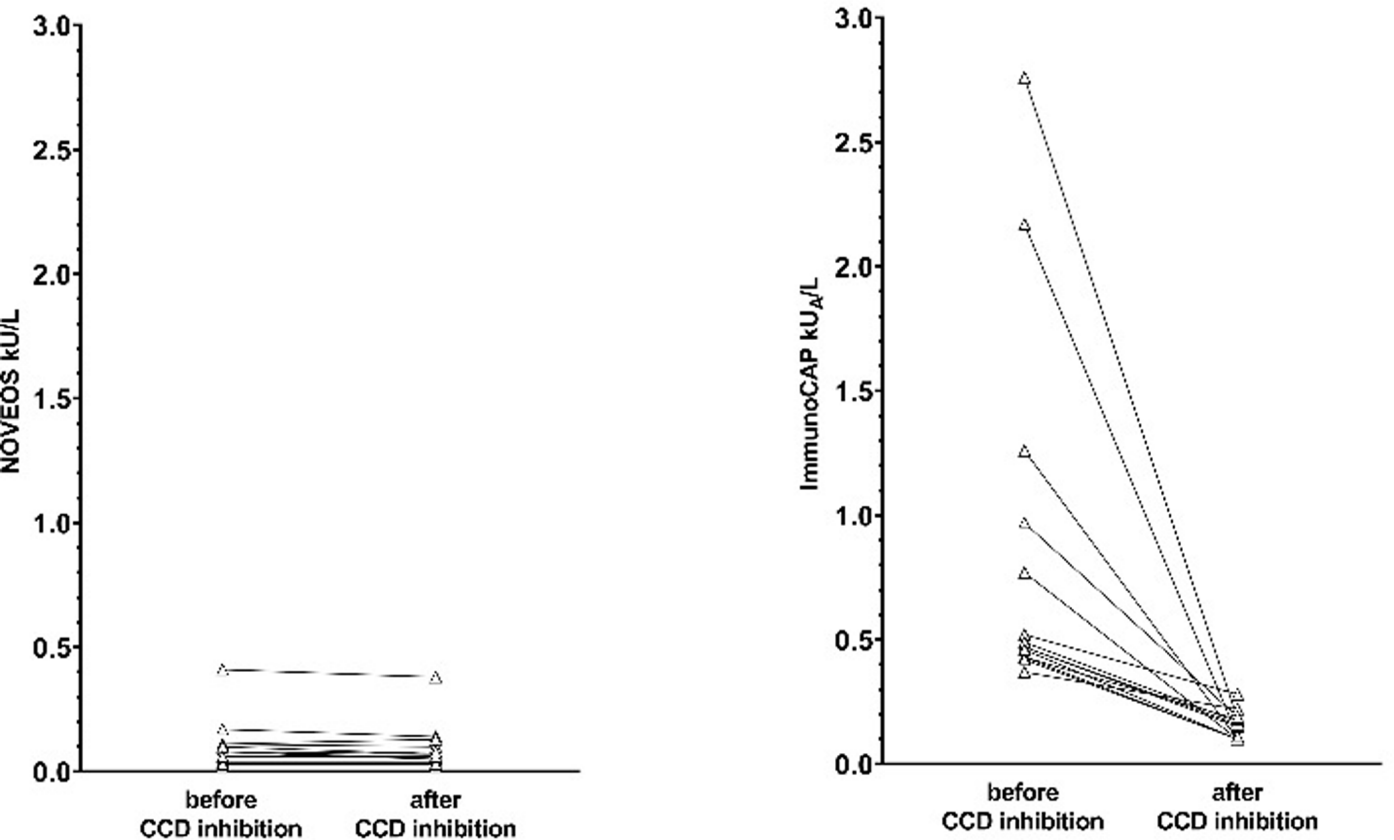
Change in specific IgE results against recombinant components before and after CCD inhibition focusing on the 14 false-positive results observed on ImmunoCAP. (a) NOVEOS Specific IgE results. (b) ImmunoCAP Specific IgE results.

In contrast, no false positive results were detected when utilizing recombinant components with NOVEOS, a non-cellulose based sIgE assay. When comparing the false-positive results that occurred on ImmunoCAP, 13 out of the 14 were negative on NOVEOS for both control and CCD inhibitor (Fig 3). There was one ImmunoCAP positive result that remained positive for NOVEOS potentially indicating a true positive sensitization. Moreover, the agreement between NOVEOS and ImmunoCAP improved when the CCD-inhibitor was utilized on ImmunoCAP resulting in a reduction of discordant results. Without the inhibitor, the total positive/negative agreement between the two assays was 82.6% (Wilson 95% CI: 73.6% - 89.0%) with a Cohen’s Kappa of 0.64 (Wald 95% CI: 0.48–0.79). This improved to 95.7% (95% CI: 89.3% - 98.3%) with a Cohen’s Kappa of 0.91 (95% CI: 0.83–1.00) when CCD inhibitor was added to the sera.

### Impact of CCD interference on low-positive specific IgE results

**Fig 4:**
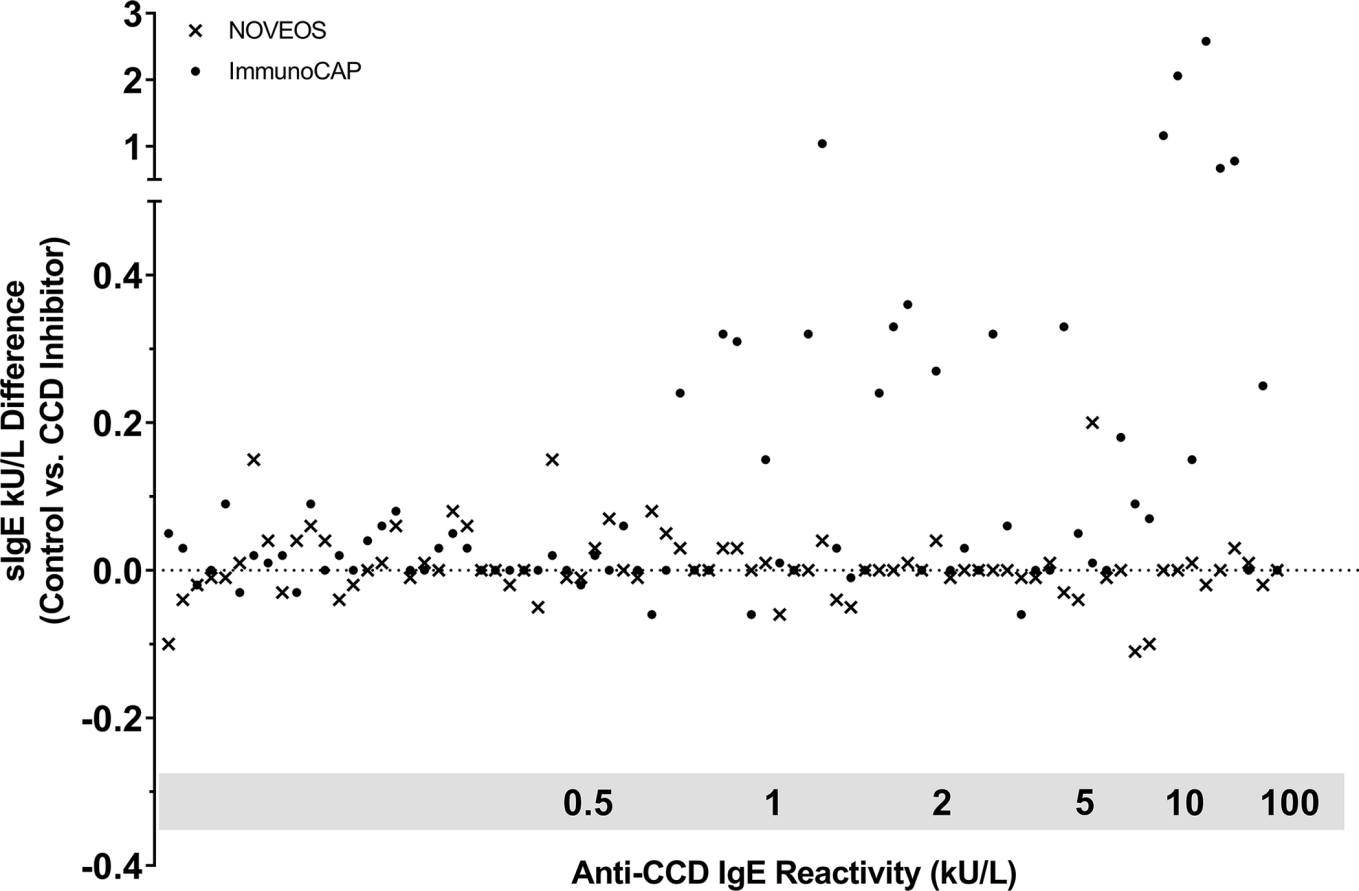
Negative to low-positive specific IgE results (< 3 kU/L) on ImmunoCAP and NOVEOS organized side-by-side in increasing CCD anti-IgE reactivity. Comparing IgE concentration difference with and without CCD inhibitor addition to the serum.

CCD-sIgE interference was mainly observed on low-positive IgE reactive results (0.35 kU/L to 3 kU/L) on ImmunoCAP (Fig 4). The resulting change in sIgE concentration with and without CCD inhibitor was significant (ANOVA, p = 0.0025) in the low-positive sample population with an average change of 27% (95% CI: 17% - 37%) compared to only 5% (95% CI: 3% - 7%) for IgE results between 3 kU/L to 100 kU/L. When the same samples were tested on NOVEOS, no significant difference were observed with and without CCD inhibitor in the low-positive sample population (average change of 8%, 95% CI: 6% - 10%).

## Discussion

In the present study, 35% of patients with known sensitization to peanut, silver birch, and/or timothy grass were positive for CCD-sIgE reactivity. Similar results have been reported in literature with prevalence rates of approximately 20–37% in allergic patients with grass pollen exposure and hymenoptera stings [8,9,13,14] and can be as high as 60% when certain pollen species are involved or when patients are sensitized to multiple pollen species.[8,21,22]

Of those patients with positive anti-CCD IgE, 17% resulted in a false positive result when tested against ImmunoCAP non-glycosylated recombinant components. Since the allergen components are free of CCD epitopes, this can only mean that CCD-sIgE is binding directly to the cellulose matrix of the allergosorbent causing inaccurate and elevated results. This was further confirmed by analyzing the same sera using streptavidin allergosorbents (absent of any allergens) on ImmunoCAP with resulting sIgE levels between 0.12 kU_A_/L to 0.65 kU_A_/L. When analyzed in a non-cellulose based assay such as NOVEOS, no false positive results were observed.

The implication of CCD interference on recombinant CRD testing is significant as it can compromise the quantitative results and thus the clinician’s ability to differentiate between genuine sensitization and cross-reactivity. As an example, in hymenoptera venom allergy, 50% of allergic patients result in double sensitization to honey bee and yellow jacket venom, due in part to CCD.[15,23–25] Recombinant CRD is able to discriminate between the rare occurrence of true double sensitization and the more common mono-sensitization [15] leading to a more accurate diagnosis and appropriate immunotherapy strategies.[26] However, in the presence of patient sera containing IgE antibodies that reacts with CCD, the diagnosis of double sensitization may remain inaccurate. With many unaware of CCD interference using recombinant CRD, clinicians may be falsely confident in such results, leading to unnecessary immunotherapy with both venoms.

Interestingly, in our study, the magnitude of CCD-sIgE in a patient sera did not necessarily correlate to a higher probability of a false-positive result. A patient with a low anti-CCD reactivity of 0.67 kU/L and another patient as high as 36.52 kU/L both demonstrated false positive results on ImmunoCAP while a patient with an even higher reactivity of 69.70 kU/L did not. The low affinity of CCD-sIgE has been repeatedly proposed in literature as a potential explanation for clinical irrelevance and variable binding; [27,28] however, recent studies have shown anti-CCD IgE can have comparable dissociation constants to those that bind to allergenic proteins. [29] Further, CCD epitopes can elicit stronger affinities when compared to other low affinity carbohydrate-binding proteins such as lectins.[29] With over 50% of sera from non-atopic blood donors containing naturally occurring IgG1 against xylose or α(1,3)-linked fucose[1,30], another explanation can be the presence of CCD specific IgG1 and IgG4 antibodies in patient sera and their ability to block the binding between CCD-sIgE and the immobilized CCD. Evidence of this potential function was observed in a study characterizing human IgE and IgG antibodies binding of N-glycans, where IgG to CCDs were observed to have markedly higher affinity compared to those binding to allergenic proteins.[29]

We propose two strategies to eliminate the risk of CCD interference in *in vitro* IgE allergy tests utilizing recombinant CRD. First, pure CCD inhibitor can be added to each serum specimen. This inhibitor should be absent of any reactive allergen protein epitope impurities and should contain several CCD structures to exploit the multivalency effect.[12] Commercial inhibitors such as ProGlycAn CCD-Blocker (www.proglycan.com, Vienna, Austria) and RIDA CCD-Inhibitor (R-Biopharm AG, Darmstadt, Germany) are readily available and meet these criteria. However, caution should be exercised when implementing CCD inhibitor as an all-encompassing solution to block the binding of CCD-specific IgE. There are rare instances in which clinical relevant CCDs are present on CCD-containing components such as native Lyc e 2 (tomato)[31] and Api g 5 (celery).[32,33] The CCD epitopes can induce mediator-released basophils from sensitized individuals while the deglycosylated form of the allergenic component cannot.

The second strategy is to utilize a non-cellulose based IgE assay to eliminate the risk of CCD interference. We demonstrate in this study that the NOVEOS IgE assay, which utilizes paramagnetic microparticles devoid of N-glycans and CCDs as an allergen carrier, is not susceptible to the same CCD interference phenomenon that is observed in the ImmunoCAP assay and Phadia systems. There are only two instances where CCD-sIgE may be detected on the NOVEOS system. The first is during the use of whole extract allergosorbents, which poses a problem for all IVD assays when CCD inhibitor is not added to the serum. The second involves native (glycosylated) component testing, which may assist in detecting clinical relevant carbohydrate epitopes. However, in regards to the context of this study and the scope of recombinant component testing, there is no risk of CCD-sIgE detection and interference using the NOVEOS system.

In conclusion, we confirm the risk of false-positive diagnostic IgE antibodies results due to CCD interference when recombinant non-glycosylated components are utilized on cellulose-based allergosorbents such as those used in the ImmunoCAP method. The incidence of CCD interference was predominantly delimited to low-positive IgE results (0.35 kU_A_/L– 3.00 kU_A_/L); however, CCD interference on the remaining results (>3kU/L) can still occur. Additionally, 17% of all positive CCD-sIgE patients, with levels as low as 0.67 kU/L, contribute to false-positive results that we observed in this study. The use of a CCD inhibitor or a non-cellulosed based assay, such as NOVEOS, can be invaluable tools for mitigating CCD-sIgE interference.

## Supporting information

S1 Fig Method comparison of NOVEOS CCD IgE assay to ImmunoCAP CCD IgE assay. Passing-Bablok regression analysis revealed a slope of 1.16 (95% CI: 0.79–1.65) and Intercept of -0.32 (95% CI: -0.75 - -0.04). NOVEOS specific IgE results are reported in kU/L and are equivalent to ImmunoCAP kU_A_/L as both autoanalyzers perform heterologous interpolation of allergen-specific IgE antibodies results from a total IgE dose response curve. NOVEOS MUXF3 CCD allergen is for investigational use only and the performance characteristics have not been established.
(TIF)

S1 Data (XLSX)
