# Supplementary figures and images for: Cross-reactive carbohydrate determinant interference in cellulose-based IgE allergy tests utilizing recombinant allergen components
Source: PLoS One. 2020 Apr 23;15(4):e0231344. doi: 10.1371/journal.pone.0231344 (PMC7179882; doi:10.1371/journal.pone.0231344)

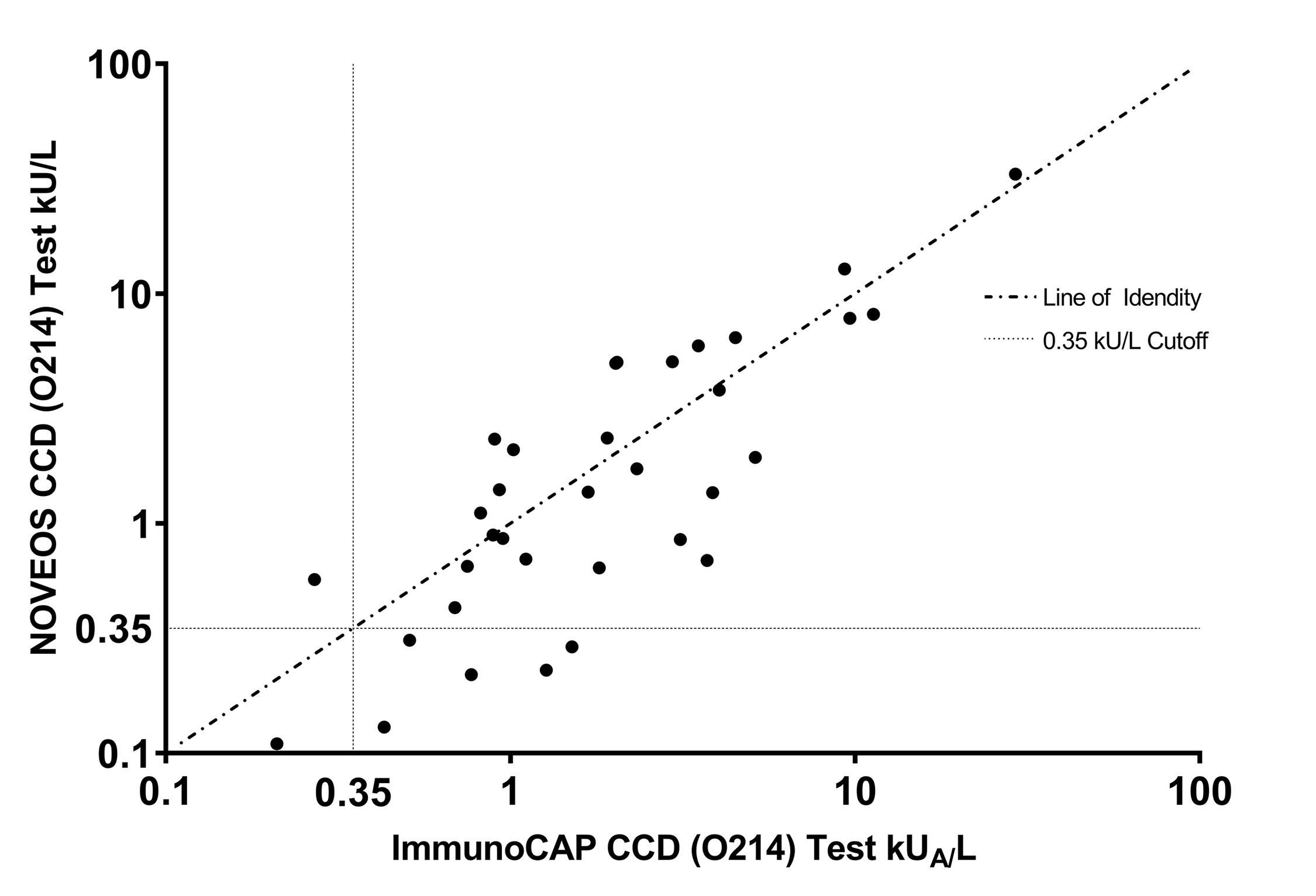

Supplement: S1 Fig — Passing-Bablok regression analysis revealed a slope of 1.16 (95% CI: 0.79–1.65) and Intercept of -0.32 (95% CI: -0.75 - -0.04). NOVEOS specific IgE results are reported in kU/L and are equivalent to ImmunoCAP kUA/L as both autoanalyzers perform heterologous interpolation of allergen-specific IgE antibodies results from a total IgE dose response curve. NOVEOS MUXF3 CCD allergen is for investigational use only and the performance characteristics have not been established. (TIF) [file pone.0231344.s001.tif]
